# What is the impact of the Rashomon approach in primary care education?: An educational case report of implementing dialogue and improvisation into medical education

**DOI:** 10.1186/s12909-021-02570-6

**Published:** 2021-03-04

**Authors:** Akiteru Takamura, Rintaro Imafuku

**Affiliations:** 1 Department of Medical Education, Kanazawa Medical University, 1-1 Uchinada-machi Daigaku, Kahoku-gun, Kanazawa, Ishikawa 920-0293 Japan; 2 Medical Education Development Center, Gifu University, Gifu, Japan

## Abstract

**Background:**

The excessive sub-divided or concrete pre-determined objectives found in the technological approach in contemporary medical education curricula may hinder the students’ spontaneous learning about diverse needs and values in care. However, medical professionals must learn the diversity for care or a variety of social factors of the patients influencing decision making in daily practice.

**Methods:**

We introduced a new method of curriculum development called the Rashomon approach. For testing the Rashomon approach, educational activities to teach the diversity in primary care were developed in four modules: 1) explication of the competency without specifying sub-objectives; 2) dialogue among multiple professional students; 3) visits and interviews of the patients; 4) dialogue with teachers’ improvisation. The students’ outcomes and responses were quantitatively and qualitatively analyzed.

**Results:**

A total of 135 medical students joined this study in 2017. The descriptive data suggested that the key concepts of diversity in primary care were fully recognized and that the pre-determined general goals were achieved. Scores on the understanding of social factors in medicine, respect for other professionals, professional identity, and satisfaction with the course were very high.

**Conclusion:**

Instead of the technological approach, the Rashomon approach, in which only a general goal guides educational activities was used in this research. Improvisation and dialogue fit the approach and were potentially effective activities to learn the multifaceted practice of medicine. In an era of competency-based education, the Rashomon approach could be a very useful framework in primary care education.

**Supplementary Information:**

The online version contains supplementary material available at 10.1186/s12909-021-02570-6.

## Background

Competency-based medical education (CBME) has become a core pedagogical paradigm to ensure that the graduates have developed the competencies required to be responsive to the needs of the communities they will serve [1–3]. Competency-based frameworks for medical education define competencies to be achieved, promoting transparency in education [4, 5]. This allows training organizations to confirm whether an educational program successfully produces graduates with the competencies needed for practice [6, 7].

However, there are also concerns in the implementation of CBME. The definition of CBME is subject to inconsistency because the term “competency” describes a rather broad concept. As the competencies, even though including broad abilities, must be incorporated CBME into their curricula, teachers must break down a competency into a number of objectives with concrete, specific and behavioral expressions; this is called the technological approach [8]. In this approach, the learning strategies and materials are systematically planned in accordance with the targeted objectives. Assessments for the objectives also need to be set according to detailed objectives [9–11]. While long lists of learning objectives across the curriculum can guide students’ learning, it becomes difficult to support individual learning needs through flexible learning plans [3]. The technological approach may not allow teachers to fully capitalize on the comprehensive nature of CBME or to encourage learners’ flexible thinking and diverse values.

The field of social medicine seeks to provide social care by understanding how social and economic conditions affect health and illness, and foster the conditions that lead to a healthier society [12]. The social determinants of health (SDOH), to which increased attention is now being paid in medical education, are a complex array of political, economic, environmental, and cultural factors that strongly influence health status and equity [12–14]. This means that modern healthcare providers need to understand that the care is not universal for all patients. Incorporating information on the SDOH into health professions curricula is challenging for teachers because the content of SDOH differs among the individuals or communities [15]. Developing effective ways of teaching variable knowledge that can adapt to the change is needed [16, 17].

Previous studies have emphasized the importance of diversity in teaching medicine, particularly in the field of primary care [18]. Physicians must respect the diversity of patient values and societal needs. Even in cases of an established diagnosis, patient care varies depending on the SDOH surrounding the patient [19]. Healthcare providers must have a high degree of competency to comprehensively understand and solve problems for each patient. As such, we must consider ways to teach the multidimensional themes of diversity and individuality in medicine. This is a limitation of the present technological approach in CBME.

There is a need to develop a new pedagogical approach to teaching the multifaceted, multi-valued themes of diversity in medicine in society. Therefore, we developed an educational program of social medicine that employs the Rashomon approach, which stresses the importance of learner subjectivity and of inductively assessing student learning through observing interactions among students, teachers, and educational resources. This study aimed to investigate how the Rashomon approach shapes medical students’ education on diversity in medicine.

### The Rashomon approach

The Rashomon approach was named after the 1950 film, *Rashomon*. In this film, a single event, a homicide is described from the different perspectives of the characters. In the Rashomon approach, teachers, like film directors, need to fully understand the big pictures so that they can engage characters = students in creative learning activities which relates to the general goals. Having a deep understanding of goals allows teachers to offer the learning activities based on their own experience and technical expertise, which is a key tenet in the Rashomon approach. In the film, flashbacks and flash-forwards were used extensively and this technique can be used to reinforce information and to stress different aspects as required [20, 21]. In the Rashomon approach, it is expected that diverse learning can be facilitated when teachers add different ideas and improvised activities to learning based on their experiences. The Rashomon approach also functions as an evaluation method for various learning of students from the activities and comprehensively assessing learners’ understanding. It is important in terms of the educational impact that unintended achievements are also emphasized in the Rashomon approach [22, 23] Therefore, in this approach, teachers must have the ability to create activities that are appropriate for the learning situation and to comprehensively assess learners. Atkin clarified the differences between the Rashomon approach and the technological approach. (see Table 1) [24–26]. In the technological approach, educational content is organized based on the teacher’s intentional educational plan. A general educational goal (Competency), which is defined as a comprehensive expression of the goals to be educated, is set first and then classified into sub-goals, which are further divided into specific objectives. To achieve these goals, teachers must plan, select and employ appropriate teaching strategies. In the technological approach, assessment requires objectivity, relevance, and credibility, making it necessary to define a generalized concept as well as certain criteria for the course evaluation.

**Table 1:** The difference between Technological approach and Rashomon approach

	Technological approach	Rashomon approach
**Developing Process**	Competency (General Educational Goals) ↓ Specific or Behavioral Objectives ↓ Developing Learning materials ↓ Implementing Learning Activities ↓ Assessment with Objectives ↓ Assessment with Competencies	Competency (General Educational Goals) ↓ Creative Teaching and Learning Activities ↓ Record or Description ↓ Assessment with Competencies
**Objectives**	Specific, Behavioral	General and non-Behavioral Or Unset
**Learning Materials**	Planned and Targeted	Discover in Learning Process
**Learning Strategies**	Planned and Targeted	Improvisation

Like the technological approach, the Rashomon approach begins with setting general goals. However, without classifying these into specific sub-goals or small objectives, teachers then generate a variety of creative teaching and learning activities on general goals, making use of their experience as experts. All events triggered by these learning activities—not only those related to the originally set general goals—should be observed and recorded [27]. In this approach, multifaceted observations of all events are incorporated into the evaluation. The effects are fed back through creative teaching and learning activities which can be achieved in an infinite number of ways.

In this respect, Rashomon’s approach is unique because it encompasses both aspects of learner-centered and teacher-led education [28]. In addition, in this approach, students learn an educational goal that includes unplanned insight from a variety of perspectives through creative learning activities with teachers.

## Methods

### Outline of educational curriculum using Rashomon approach (see Table 2)

#### Setting

Medical schools in Japan are six-year undergraduate programs. In the 2016 revision of the core curriculum for medical schools in Japan, the Ministry of Education, Culture, Sports, Science and Technology (MEXT) emphasized the importance of social medicine in medical education [29]. Traditionally, Kanazawa Medical University has offered classes in third-year medical students on diversity in medicine with a didactic approach, in a series of lectures. However, from the feedback from students after the classes, it could not be said that it was not sufficiently learned. We developed a new curriculum and Third-year medical students at Kanazawa Medical University joined the learning session.

**Table 2:** Outline of Social Medicine Learning based on Rashomon Approach

Rashomon approach	Social Medicine Learning Session
General Educational Goals (≒Overall Competency)	“Understand the relationship between society and health / diseases and learn the impact on health of individuals and social life due to changes in environmental factors surrounding individuals and group of people.”
• Creative Teaching and Learning Activities “Discover the value of learning material in learning process” “Emphasis on improvisation” • Record or Description of Learning	◆ Developing teaching and learning activities based on creative task interpretation · Module 1: Explication of the overall competencies · Module 2: Dialogue among students · Module 3: Visits a patient’s home and interviews · Module 4: Dialogue with teachers’ improvisation ◆ Faculty’s Competencies · As unexpected lesson could be developed, it might be fun but tense for faculty. · It emphasizes the competence of the faculty as professionals.
• Assessment with Competencies	◆ Assessing the descriptive data (written data) from the students (What they learnt and what they understand)

### Learning strategy

#### Interprofessional patient home visits and dialogue

We developed a social medicine program consisting of four modules. We offered an improvisation activity to facilitate peer-to-peer and teacher-to-student dialogue from multifaceted viewpoints. The students were divided into groups comprising medical students, nursing students, and pharmaceutical sciences students. The first module was an interactive lecture-based four-hour introduction class to clarify the general goals suggested by MEXT. These general goals were as follows: Understand the relationship between society and health/diseases and Learn the impact of changes in environmental factors surrounding individuals and groups of people on their health and social lives [29]. The students were asked to investigate the issues for the general goals and identify factors influencing patients’ social lives and health. The second module was an interactive team-based four-hour dialogue session. Each group decided in detail what information to collect from the patient based on the general goals suggested by MEXT. As the group members were from different professional fields, they were able to come up with ideas from their own field’s point of view. The third module consisted of patient’s home visits over a three-day period. Students visited the patients’ homes and attempted to pursue the key inquiries discussed in Modules 1 and 2 through conversation with the patient. In the fourth module, there was another dialogue session with the students and three teachers who are a doctor, a nurse, and a pharmacist, have over 10 years’ clinical experience in communities. During this session, the students presented their experiences at the patient’s home. The teachers added impromptu explanations to go along with the students’ answers and participated in the dialogue with the students. They asked relevant questions, and shared their ideas and experiences to help the students deepen their understanding of the gathered information. Because these improvised responses based on the teachers’ clinical experience were intended solely to facilitate the learners’ understanding, the teachers never sought predetermined answers to their questions or led the discussion in a way that students would find particular solutions to the problems they presented during the session.

### Data collection

To clarify how and what students learned through the sessions based on the Rashomon approach, we developed a feedback survey to assess the utility of the curriculum and to determine which concepts the students perceived as most beneficial after completing the four modules [30, 31]. A questionnaire and a survey were designed to understand the strengths and the weakness of the curriculum. The questionnaire consisted of five questions that asked about the content of this curriculum. The questions were about students’ understanding of the general goals and their usefulness in practice, the importance of respecting other professions, the establishment of self-identity, and the enjoyment of the session itself (Additional file 1). Medical students ranked their responses on a five-point Likert scale as follows: 1 = worst, 2 = not good, 3 = neutral (neither not good nor good), 4 = good, 5 = best. The survey consisted of two semi-structured questions that specifically asked what students had learned through the session and what the structure of the session had been. These were written as free statements (Additional file 2). To preserve anonymity, identifying information was not requested.

### Data analysis

For the quantitative data analysis, we used descriptive statistics to analyze the students’ responses to the questionnaire about the learning modules. Statistical analyses were performed using IBM SPSS Statistics for Windows, Version 20 (IBM Corp., Armonk, NY, USA). For the qualitative data analysis, all survey answers were reviewed to identify keywords and concepts for coding. From this, we created an initial thematic map. Using the thematic analysis method, we then defined and named the final themes for analysis to develop a final thematic map [32]. To validate the constructs, preliminary results were shared with other members of the teaching staff in the same department who were not otherwise involved in the analysis. This study was approved by the Kanazawa Medical University Review Board. IRB number is I-090.

## Results

### Student evaluation scores reported in the questionnaire (see Table 3)

A total of 135 medical students who completed this course responded to the survey. Their scores of the modules’ usefulness were very high overall. The students’ mean (SD) usefulness evaluation scores were 4.4 (0.4) for understanding social determinants of health, 4.4 (0.57) for future practice, 4.5 (0.54) for respecting other professionals, 4.5 (0.50) for their own self-identity as a doctor, and 4.3 (0.45) enjoyableness.

**Table 3:** The results of student evaluation scores of the course. On a scale of 1-5 with 5 being “very much”

Question / Scale	1	2	3	4	5	Average Score
Understanding social determinants of health, n	0	0	0	82	53	4.4±0.49
Usefulness for future practice, n	0	2	0	76	57	4.4±0.57
Respecting other professionals, n	0	1	0	70	64	4.5±0.54
Increasing Self-identity as a doctor, n	0	0	0	67	68	4.5±0.50
Enjoyable, n	0	3	2	60	70	4.3±0.45

### Student learning themes (see Table 4)

Five themes were extracted from the students’ descriptive data: 1) Comprehensive background of patients; 2) Multiple points of view from different types of a health professional; 3) Shared decision making with patients; 4) Many uncertain factors in medicine; and 5) Different patients’ attitudes at patients’ own places.

**Table 4:** Theme of the students’ learning and students’ comments

・What have you learned in this social medicine curriculum? ・How was the learning session with the lecturer?	
Theme	Example of Medical Students’ Comment
Comprehensive background of patients	Student A “As a result of having a good relationship with sufficient preparation in advance and enough time at the patient's home, I was able to hear about the patient's life and thoughts.”
Multi health professionals’ points of view	Student B “I have been able to know the patient from various aspects because I got many viewpoints from other professional students that I do not notice, such as the medicine and the things that the patients should be aware of in their daily life.”
Shared decision making with patients	Student C “The patient had a strong will for future life-prolonging treatment. Since it was completely different from my thoughts, different people had different values and I felt that I had to discuss and decide.”
Many uncertain factors of medicine	Student D “I have studied biologically clinical reasoning, diagnosis, and treatment in the class, but I understood that when it comes to real practice, I have to consider not only the patient's values but also the economic and family conditions.”
Different patients’ attitudes at patients’ own places	Student E “I noticed a difference in the facial expression of the patient at home compared to the university hospital. I thought it was important to check the various situations at home because there were a lot of things I didn't know in the consultation room at the hospital as to what kind of life the patients have and how they have a relationship with my family.”

### Themes about the curriculum development with Rashomon approach (see Table 5)

Similarly, an additional five themes were extracted from student descriptive data that seemed to be not directly related to educational goals but were considered important for curriculum development: 1) The importance of and interest in self-directed learning; 2) A framework for information provided to students based on teachers’ experience; 3) Different perspectives and values among health professionals; 4) Having fun engaging in dialogue and naturally understanding topics; and 5) Anxiety regarding assessment.

**Table 5:** Theme about the curriculum development with Rashomon Approach from the students’ comments

Other Important Themes on Learning	
Theme	Example of Medical Students’ Comment
The importance of and interest in self-directed learning	Student F “We have learned what teachers teach in class, but when there are a lot of factors we have to take into account, there are limits to teaching, and I felt it was important to learn in people who have a variety of values.”
A framework for student information provided by the lecturers’ experience	Student G “The teacher's experiences helped me to understand, but the teacher's values also have a big impact on students.”
Different perspectives and values among health professionals	Student H “It was easy for me to understand that we had different views from pharmacy students and nursing students, but I was surprised that the view was very different even among the medical students. I realized that doctors may have their own way of thinking about treatment policy.”
Fun of dialogue and naturally understanding topics	Student I “Everyone had a natural and enjoyable understanding through the dialogue on a large theme rather than just learn what was decided.”
Anxiety regarding assessment	Student J “I am worried about how my assessment will be done because there are no clear learning objectives and no criteria for the assessment.”

## Discussion

This study has investigated how the Rashomon approach shapes medical students’ learning of the SDOH in primary care settings [33, 34]. At the beginning of the program, we presented general goals for learning to students. These goals offered just a framework for the students to better understand patient diversity. However, we intentionally did not set specific objectives ahead of time. This approach allowed us to support student-centered active learning. The absence of specific objectives was particularly important in the planning stage, when students’ groups discussed and identified the information that should be elicited from the patients.

Student groups visited patients’ homes rather see the patients at the university hospital. This created an environment where patients could more openly share their opinions with the students. Moreover, our findings show that this approach allowed students to gain multidimensional real-world experience in primary care they would engage in as doctors. Indeed, three themes (comprehensive backgrounds of patients, different patients’ attitudes and patients’ own places, and shared decision making with patients) indicated that the students experienced the real lives of patients who might have found it easier to share their stories outside the hospital context during the patient home visits.

The spread of evidence-based medicine has advanced the standardization of disease treatment policies. On the other hand, health professionals often play a role in solving patient social and psychological problems that include complex, multifaceted issues. Although teaching uncertainty and diversity in care is challenging, the students perceived that they had learned about the many uncertain factors in medicine through the modules. The results suggest that the students were able to understand the various perspectives of the patients and different health professionals.

In the field of medical education, assessing students’ achievement of comprehensive competency in programs with competency-based curricula has become a significant challenge [3]. The broad nature of competencies complicates the assessment of students’ achievements. As a result of fragmenting general competencies into numerous sub-goals, the competencies learners are actually able to acquire may be limited to those that were explicitly taught. To overcome this problem, our newly developed curriculum uses the Rashomon approach to provide a useful framework for assessing students’ achievements from diverse perspectives [24, 35–37]. A better understanding of social medicine was set as a general goal, but the additional objectives that students achieved were not detailed in advance in this program. We think that this resulted in the broader achievement of the competency.

In accordance with the Rashomon approach, students were encouraged to engage in a deep dialogue amongst themselves concerning many elements of society based on their experience visiting a patient’s home with their interprofessional student groups. In a dialogue, two individuals work with and challenge each other to collaboratively alter and deepen their understanding [38, 39]. Additionally, Bohm et al. stated that “no firm rules can be laid down for conducting a dialogue because its essence is learning” [40]. For this dialogue, teachers did not provide any instructions or learning strategies. Following the students’ home visits, there was also a dialogue session between teachers and students. In the student–teacher dialogue, the faculty responded extemporaneously to student achievements and questions and refrained from evaluation through ranking, assigning scores, or completing checklists. This reflects the nature of dialogue and of the Rashomon approach [41].

However, it was still necessary to verify how much students deepened their understanding of the SDOH through this learning approach. Table 4 shows that students autonomously understood medical uncertainties and diversities, such as patient values, views on life, and other SDOH. Students described economic factors, cultural factors, the social environment, and even the importance of social networks. As mentioned above, it was likely easier for the patients to talk to the students about their social backgrounds in their own homes, reducing the cognitive load of this task [42]. It also may have been easier for the students to understand the contextual information gained by visiting the patients’ homes through situated learning [43]. Using the information provided by the patients about polypharmacy and multimorbidity, the students came to understand the importance of shared care responsibilities and coordination between health professionals. In terms of patients’ values, the students mentioned the importance of sharing their opinions and engaging in the decision-making process as a team including patients. Through dialogue with the patients in their places and through dialogue with faculty members in the classroom context, the students came to understand uncertainty and diversity in care, which can be seen as practicing social constructivism [44].

The results indicated that the newly developed curriculum using the Rashomon approach had a positive impact on the students’ learning. Based on students’ comments on some of the educational aspects of the Rashomon approach, they were able to understand the role of social factors and diversity in medicine. In particular, the dialogues provided a framework for the students’ free and multi-valued learning. Teaching clinical problems with specific solutions in medicine is important, but it is also essential for students to develop the competency to manage problems with uncertain answers in daily practice.

Overall, the interprofessional students’ learning with the Rashomon approach seems useful for students. Though in modern medical education, it is common to develop curricula with clear and measurable achievement goals, things which thought to be important but unwritten in the goals are often tend to be ignored. Clinical facts that the students should learn in the real world involves a variety of uncertainties and multiple aspects. This approach may be able to solve those issues. Activities involving teachers’ improvisation encourages students to actively participate in learning and to gain a better understanding of diversity in medicine. Learner-centered education is important, but teacher-led education is also key in the Rashomon approach. SDOH education following the Rashomon approach provides an opportunity for students to learn how to effectively assess and address the social factors and difficulties that commonly accompany patients’ biomedical concerns. It is important to set up a learning environment that presents a comprehensive set of general goals without sub-dividing objectives like the technological approach and that freely considers diverse values. Although the Rashomon approach appears to be generally effective, the areas in which the Rashomon approach is more effective for students’ learning and the areas in which the technological approach is more effective remain unclear. In this case, we set the condition that students of various professional backgrounds participated and that part of the program was implemented at patients’ homes. In addition, the improvisational skills of the teachers and the dialogues among students and with teachers played an important role in the sessions. While these conditions may have contributed significantly to the positive results in this study, the general conditions in which the Rashomon approach is more effective have not been established. However, there seem to be some limitations. Though we assessed the students’ competency of understanding the diversity and the uncertainty in medicine by use of the questionnaire and the free-writing documents, this may not reflex their true achievement. To validate this approach, we need to measure the change of the students’ behavior and performance in the future. In addition, the role of teachers is the most important key in this approach, and the achievement level of students may be influenced by the experience and values of teachers who lead the improvisational dialogue. Therefore, teacher training remains a challenge. And additional research is needed to determine under what conditions and in what domains to adopt the Rashomon approach.

This study demonstrates that students benefit from improvisation activities led by teachers and from dialogue among students and teachers in a program whose curriculum underpinned by the Rashomon approach. The Rashomon approach specifically focuses on facilitating students’ understanding of diversity through creative learning activities. For learning variable factors such as SDOH, curriculum development using the Rashomon approach seems to work, especially in primary care education.

## Supplementary Information

**Additional file 1.** The questions in the survey questionnaire.

**Additional file 2.** Social medicine survey questions.

## Data Availability

The datasets used and analysed during the current study are available from the corresponding author on reasonable request.

## Abbreviations

CBME: Competency-based medical education
SDOH: social determinants of health
MEXT: the Ministry of Education, Culture, Sports, Science and Technology in Japan
