# Supplementary material for: What is the impact of the Rashomon approach in primary care education?: An educational case report of implementing dialogue and improvisation into medical education
Source: BMC Med Educ. 2021 Mar 4;21:143. doi: 10.1186/s12909-021-02570-6 (PMC7934433; doi:10.1186/s12909-021-02570-6)
Supplement: Supplementary file 1 — Additional file 1. The questions in the survey questionnaire. [file 12909_2021_2570_MOESM1_ESM.docx]

**Additional file 1: The questions in the survey questionnaire**

| Q1: Did you understand the general goals (mainly Social determinants of health) through the session? |
| --- |
| Q2: Do you think that the session is useful for future practice? |
| Q3: Do you think respecting other professionals is important through the session? |
| Q4: Was your self-identity as a doctor increased with other professional students? |
| Q5: Do you think that this session was enjoyable? |
