# Supplementary material for: What is the impact of the Rashomon approach in primary care education?: An educational case report of implementing dialogue and improvisation into medical education
Source: BMC Med Educ. 2021 Mar 4;21:143. doi: 10.1186/s12909-021-02570-6 (PMC7934433; doi:10.1186/s12909-021-02570-6)
Supplement: Supplementary file 2 — Additional file 2. Social medicine survey questions. [file 12909_2021_2570_MOESM2_ESM.docx]

**Additional file 2: Social medicine survey questions**

| **Question** | **Response Type** |
| --- | --- |
| Q1: What have you learned in this social medicine curriculum?  Q2: How was the learning session with the lecturer? | Open answer (Description) |
| Additional comments about the learning module | Open answer (Description) |
